# Contributing to women’s health nursing by disseminating research

**DOI:** 10.4069/whn.2024.12.16.1

**Published:** 2024-12-31

**Authors:** Sue Kim

**Affiliations:** College of Nursing, Mo-Im Kim Nursing Research Institute, Yonsei University, Seoul, Korea

The year 2024 marks a year of significant developments for the journal. First of all, the title was changed to *Women’s Health Nursing* (WHN) from *Korean Journal of Women Health Nursing*. This change was instituted to commemorate the journal’s indexing in MEDLINE in 2023 [1], an honor that is bestowed to roughly only one-tenth of journals that apply to MEDLINE [2]. The new title also reflects our commitment to reaching a global audience, extending the journal’s scholarly contributions beyond Korea and Asia to authors worldwide. Additionally, this year, Clarivate made the policy decision to assign an impact factor (IF) to all journals in the Web of Science Core Collection, starting with the 2023 Journal Citation Reports [3]. As an Emerging Sources Citation Index (ESCI) journal, we obtained a 2023 IF of 1.0, matching the IF of the *Journal of Korean Academy of Nursing*, a well-regarded Korean SCIE (Science Citation Index Expanded) journal. Finally, this year is also notable as it marks the 30th anniversary of the Korean Society of Women Health Nursing, for which WHN is the official journal. In Korea, a 30-year period is referred to as “*yi-rib*” (이립, 而立), meaning “a time of establishing all foundations” according to Confucian traditions. Our academic society has indeed made significant progress and laid a solid foundation for women’s health nursing in Korea. As the journal published its first article in 1995, we are nearing our own 30-year milestone.

Considering the developments of this year and looking forward to 2025, WHN is more committed than ever to disseminating women’s health research for nurses in academia and practice. We recognize the serious responsibility this entails and approach it with great eagerness. We will continue to critically assess and disseminate quality articles that contribute to women’s health nursing, such as the paper in this issue which calls for a critical awareness of gender and its integration into nursing research [4]. As 2025 marks the 30th anniversary of the journal, we plan to publish a special issue titled “Digital Health Interventions (DHI) for Women’s Health” in June 2025. This issue will serve as a timely follow-up to our 2023 special issue on “Digital Era Education for Women’s Health and Wellbeing” (https://www.kjwhn.org/upload/pdf/kjwhn_v29n3_full.pdf), which explored AI-based healthcare education opportunities and challenges [5]. Given the rapid increase in DHI and their recognized value for improving primary healthcare [6] and preventing deaths [7], exploring how DHI can promote women's health will be invaluable. The 2025 special issue on DHI will cover topics such as digital devices (wearable or other tracking modalities), remote digital monitoring, artificial intelligence, and mobile apps, all applied as interventions for women’s health and wellbeing. We welcome submissions including systematic reviews, scoping reviews, methodology papers, concept papers, and experimental research to clarify the state-of-the-science, as well as qualitative and mixed-method research to provide deeper insights.

## Journal metrics

Each year, we compile journal metrics and evaluate the journal's processes and outcomes. As shown in Table 1, WHN received a total of 117 manuscript submissions this year, including 102 unsolicited manuscripts. This marks a continued increase in unsolicited submissions, up from 80 in 2023 [8] and 63 in 2022 [9]. Of these unsolicited manuscripts, 59 (57.8%) were from international authors, indicating the global impact of our indexing in PubMed Central and ESCI in 2022. However, it is important to note an increase in unsuitable manuscripts: 57 in 2024, compared to 30 in 2023 [8]. These unsuitable submissions, typically desk rejections due to misalignment with the journal’s aims and scope, underscore the importance for prospective authors to thoroughly review WHN’s aims and scope before submitting their manuscripts.

## Appreciation for 2024 reviewers

I wish to acknowledge the following dedicated reviewers who have supported WHN this year:

Ahn, Suk Hee (Chungnam National University)

Bae, Kyungeui (Dongseo University)

Chae, Hyun Ju (Joongbu University)

Chae, Myung-Ock (Cheongju University)

Choi, Mi Jin (Chodang University)

Choi, Mi Young (National Evidence-based Healthcare Collaboration Agency)

Chung, Chae Weon (Seoul National University)

Chung, Mi Young (SunMoon University)

Emily E Drake (University of Virginia)

Go, Gee Youn (Dongguk University Ilsan Hospital)

Ha, Ju Young (Pusan National University)

Han, Jeehee (Chung-Ang University)

Jang, Hyun-Jung (Catholic Kkottongnae University)

Jeong, Geum Hee (Hallym University)

Jun, Eun-Young (Daejeon University)

Kang, Sookjung (Ewha Womans University)

Kim, Eun Jin (Woosuk University)

Kim, Haewon (Seoul National University)

Kim, Hye Young (Keimyung University)

Kim, Hyun Kyoung (Kongju National University)

Kim, Jeung-Im (Soonchunhyang University)

Kim, Kyungwon (Daegu Haany University)

Kim, Mi Jong (Hannam University)

Kim, Miok (Dankook University)

Kim, Moonjeong (Pukyong National University)

Kim, Myoung-Hee (Semyung University)

Kim, Su Hyun (Nambu University)

Kim, Sue (Yonsei University)

Kim, Sun-Hee (Daegu Catholic University)

Kim, Sun Ho (Chungbuk National University)

Kim, Yeun Mi (Suwon Women’s University)

Kim, Yoonjung (Hallym University)

Kim, YoungJu (Daejeon Health Institute of Technology)

Ko, Eun (Sunchon National University)

Kwon, Yu Rim (Ansan University)

Lee, Ju-Young (Catholic University)

Lee, Sun-Kyoung (Seoul Women’s College of Nursing)

Nho, Ju Hee (Jeonbuk National University)

Park, Seo A (Gyeongbuk College of Health)

Park, So Mi (Yonsei University Wonju)

Seo, Minjeong (Gyeongsang National University)

Shin, Gi Soo (Chung-Ang University)

Song, Ju-Eun (Ajou University)

Song, Young A (Ansan University)

Yeom, Gyejeong (JEI University)

Yoon, Ji Won (Shinhan University)

As “Reviewers of the Year 2024,” the journal congratulates the following reviewers for their dedication and sharp eyes:

• Mijong Kim (Hannam University)

• Hyunju Chae (Sangmyung University)

• Kyungwon Kim (Daegu Haany University)

Special recognition also goes to our “Editor’s Pick 2024 Award,” as the most cited manuscripts for the period of 2022–2023 in the Scopus and Web of Science databases.

• Hwang WY, Choi SY, An HJ. Concept analysis of transition to motherhood: a methodological study. Korean J Women Health Nurs. 2022;28(1):8-17. https://doi.org/10.4069/kjwhn.2022.01.04

• Jung S. Challenges for future directions for artificial intelligence integrated nursing simulation education. Korean J Women Health Nurs. 2023;29(3):239-242. https://doi.org/10.4069/kjwhn.2023.09.06.1

WHN has been laying the groundwork for 29 years and is now poised to cross the threshold into “yi-rib.” We invite you to join us in making a significant impact on women’s health by sharing your research and contributing to the advancement of women’s health nursing.

## Figures and Tables

**Table 1.** Basic statistics on manuscripts submitted to *Women’s Health Nursing* in 2024

Category	Data	Notes
Commissioned manuscripts (n)	13	5 editorials, 4 issues and perspectives, 4 invited papers
Unsolicited manuscripts (n)	102	
Accepted manuscripts (n)	35	
Non-accepted manuscripts (n)	69	7 rejected after review, 59 rejected before review (unsuitable), 3 withdrawn
Manuscripts reviewed and decided upon (n)	42	35 accepted, 7 rejected
Manuscripts under review or revision (n)	15	
Acceptance rate (%)	61	35/57=0.61
